# Environmental influences on sinking rates and distributions of transparent exopolymer particles after a typhoon surge at the Western Pacific

**DOI:** 10.1038/s41598-021-88477-0

**Published:** 2021-05-31

**Authors:** M. Shahanul Islam, Jun Sun, Guicheng Zhang, Zhuo Chen, Hui Zhou

**Affiliations:** 1 College of Food Engineering and Biotechnology, Tianjin University of Science and Technology University, TEDA, No 29, 13thAvenue, Tianjin, China; 2 College of Marine Science and Technology, China University of Geosciences (Wuhan), Wuhan, 430074 China; 3 Research Centre for Indian Ocean Ecosystem, Tianjin University of Science and Technology, Tianjin, 300457 China; 4 Key Laboratory of Ocean Circulation and Waves, and Institute of Oceanology, Chinese Academy of Sciences, Qingdao Collaborative Innovation Center of Marine Science and Technology, Qingdao, 266071 China

**Keywords:** Carbon cycle, Marine biology

## Abstract

A multidisciplinary approach was used to investigate the causes of the distributions and sinking rates of transparent exopolymer particles (TEPs) during the period of September–October (2017) in the Western Pacific Ocean (WPO); the study period was closely dated to a northwest typhoon surge. The present study discussed the impact of biogeophysical features on TEPs and their sinking rates (sTEP) at depths of 0–150 m. During the study, the concentration of TEPs was found to be higher in areas adjacent to the Kuroshio current and in the bottom water layer of the Mindanao upwelling zone due to the widespread distribution of cyanobacteria, i.e., *Trichodesmium hildebrandti* and *T. theibauti*. The positive significant regressions of TEP concentrations with Chl-a contents in eddy-driven areas (R^2^ = 0.73, especially at 100 m (R^2^ = 0.75)) support this hypothesis. However, low TEP concentrations and TEPs were observed at mixed layer depths (MLDs) in the upwelling zone (Mindanao). Conversely, high TEP concentrations and high sTEP were found at the bottom of the downwelling zone (Halmahera). The geophysical directions of eddies may have caused these conditions. In demonstrating these relations, the average interpretation showed the negative linearity of TEP concentrations with TEPs (R^2^ = 0.41 ~ 0.65) at such eddies. Additionally, regression curves (R^2^ = 0.78) indicated that atmospheric pressure played a key role in the changes in TEPs throughout the study area. Diatoms and cyanobacteria also curved the TEPs significantly (R^2^ = 0.5, P < 0.05) at the surface of the WPO. This study also revealed that TEP concentration contributes less to the average particulate organic carbon in this oligotrophic WPO.

## Introduction

Generally, polysaccharide-based transparent exopolymer particles (TEPs) are derived from microorganisms, i.e., phytoplankton^1–5^, depending on their physiological state^6,7^ and bloom condition^4,8^. This secretion is also influenced by various environmental conditions, i.e., chlorophyll-*a* (chl*-a*)^9^, nutrient content^10^, salinity^11^, turbulence^12,13^ and CO_2_ concentration in the water^14^. However, the production of TEPs associated with phytoplankton mainly depends on the concentrations of diatoms^15,16^ and cyanobacteria^17^. As an oil droplet^1^, TEP secretion is a defense mechanism of phytoplankton; these secretions aggregate at the surface^3^ of the ocean and contribute to atmospheric carbon by bubble bursting or wave action^4^. In addition, TEPs occasionally act as food sources for zooplankton across the ocean^17,18^.

Complex correspondences of TEPs with phytoplankton and environmental conditions were reviewed in previous studies^19^. They reported that the relation between phytoplankton compositions and oceanographic processes is complex through the western boundary currents^20^. For example, phytoplankton blooms have been reported on currents that drive upwelling^21^, which may amplify TEPs along adjacent areas^4,8^. However, TEPs have been reported to be higher in coastal areas^22,23^, especially in estuaries^24,25^, rather than in the open ocean^26–29^. More specifically, high TEPs have been found at mixed layer depths (MLDs) in the ocean due to their ability to stick to each other^4,30^. It was also reported that the TEP concentrations^28^ and sinking rate (sTEP)^24^ were higher in the surface current active zone of marine environments. Beside current activities, Low sTEP may affect by TEPs for its stickiness^8,19^. Therefore, local geophysical features may influence the concentration and sinking rates of TEPs through external forcing. Likewise, TEPs are also hypothesized to be affected indirectly or actively by eddies and oceanic circulations. Considering these phenomena, the Western Pacific Ocean (WPO) is a suitable study area, as it hosts numerous circulations and eddies^31^.

**Figure 1:**
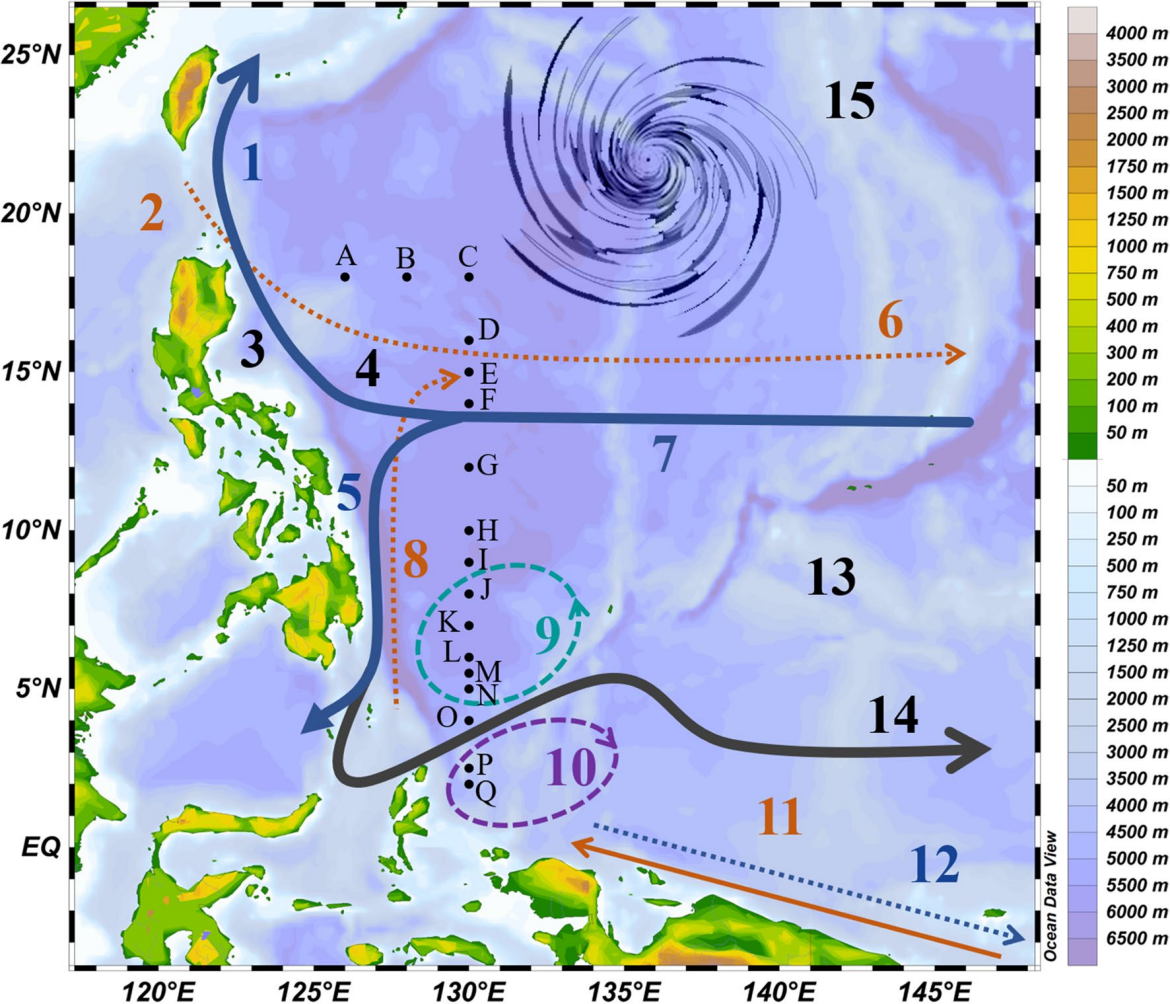
Sampling stations (St. A–Q) with different local currents (A), i.e., 7 = North Equatorial current (NEC), 6 = North Equatorial under current (NEUC), 14 = North Equatorial counter current (NECC), 1 = Kuroshio current (KC), 2 = Luzon Under current (LUC), 5 = Mindanao current (MC), 8 = Mindanao under current (MUC), 11 = New Guinea coastal current (NGCC), and 12 = New Guinea coastal under current (NGCUC), with the related geo-physical forcings, i.e., 9 = Mindanao Eddy (ME), 10 = Halmahera Eddy (HE), 3 = Philippine trenches (PT) in the Western Pacific Ocean (WPO). Additionally, Typhoon Lan^45^ occurred near the Philippine coast during the sampling period in the WPO (13).

Open ocean currents, particularly the boundary currents of the WPO, are referred to as unproductive zones compared to the Eastern Pacific Ocean (EPO) boundary currents^20^ and coastal areas^32–35^, as they displace the upper layer of productive waters, mostly in polar regions^20,36,37^. The WPO water column (Fig. 1) is influenced by various currents^31^, i.e., the North Equatorial current (NEC), North Equatorial undercurrent (NEUC), North Equatorial counter current (NECC), Kuroshio current (KC), Luzon undercurrent (LUC), Mindanao current (MC), Mindanao undercurrent (MUC), New Guinea coastal current (NGCC), and New Guinea coastal undercurrent (NGCUC); vortices^38,39^, i.e., the Mindanao Eddy (ME) and Halmahera Eddy (HE); and local geological features, i.e., the Philippine trench (PT), Philippine Basin (PB), and Kyushu Palau Ridge (KPR), which influence species biodiversity, nutrient distribution and particles transportation^40,41^.

Previously, western boundary currents have been reported as intensive circulations of nutrients that may directly affect the local phytoplankton community^42^ and occurrence of particle sinking^20,24,37^. However, the effect of ocean water circulations (currents, eddies, etc.) on TEPs has remained unclear. The present study was designed to determine the effects of biological parameters influenced by ocean circulations on the distribution of TEPs^43^ and sTEP, as well as the associated carbon concentrations (TEP_C_), at different depths of the WPO^44^. The present study was conducted 4 days after the attack of super typhoon (category 4) Lan^45^, which may have also locally affected the TEP distribution (Fig. 1). To uncover its causes and effects, correlations between TEPs and sTEP with other environmental parameters will be investigated after considering WPO eddies and current patterns accordingly.

## Results

### Western pacific hydrology

**Figure 2:**
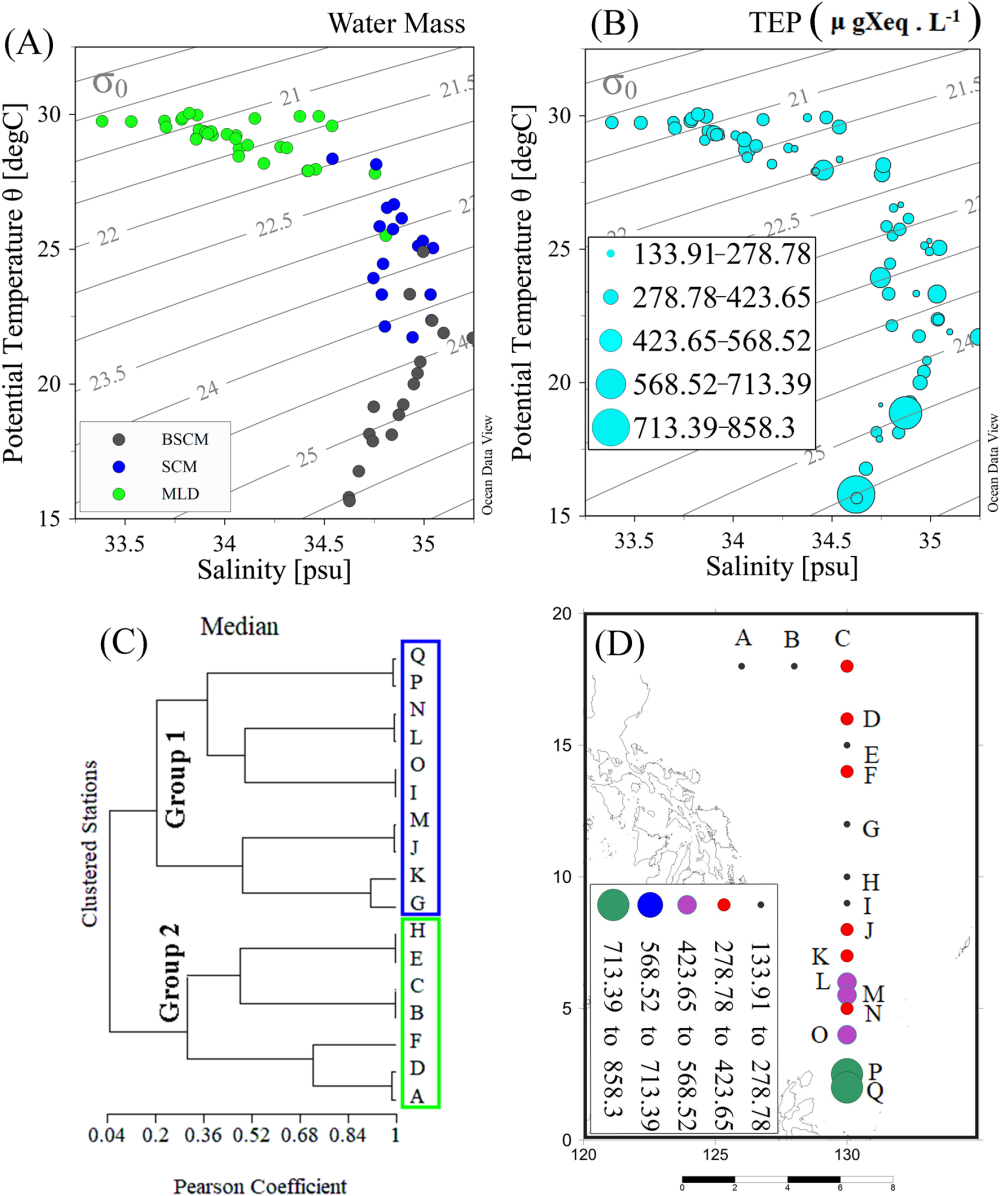
TS diagraph of samples with water density gradients (**A**) compared to TEP concentrations along those water masses (**B**). Cluster analysis of all stations considering nutrients as variables (**C**) and average TEP concentrations (**D**) at the WPO.

The vertical zonation of the Western Pacific water column and its features are important to understanding current findings. Previously, WPO water masses were categorized into different sections^46^, i.e., North Pacific tropical water (NPTW) near 11° N^47,48^, North Pacific intermediate water (NPIW) near 7° N^49,50^, North Pacific tropical subsurface water (TSSW) between the NPTW and SPTW^51^, South Pacific tropical water (SPTW) and Antarctic intermediate water (AAIW) near 1° N^52–56^, with an intertropical convergence zone (ITCZ) between 4°–8° N^57^. Most of the study stations were aligned on the gridline of 130° E (Fig. 1), covering a depth of 0–150 m, which included the subsurface chlorophyll maximum (SCM) layer. Therefore, this transect was divided into three vertical water layers^27^, i.e., the MLD (0–50 m), SCM (50–100 m) and depths below the SCM (BSCM; 100–150 m) rather than two layers, as reported in previous studies^58^. These segmentations were also supported by T-S clustering of the sampling stations into three groups (Fig. 2A), which may further explain the vertical congregations of TEPs (Fig. 2B).

### Geo-physical zonation of stations

**Figure 3:**
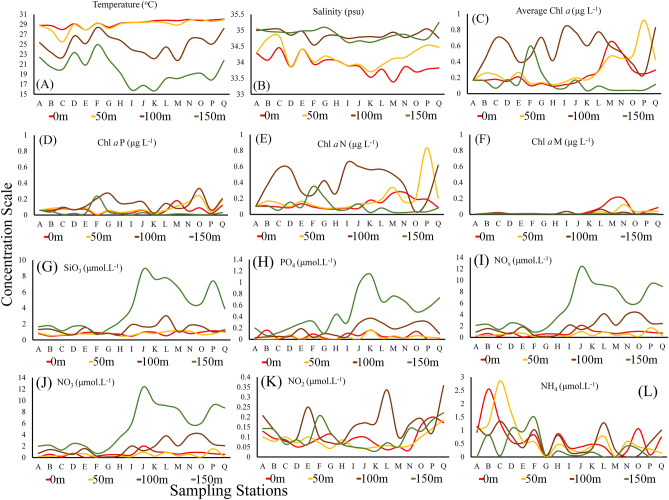
Different environmental parameters, i.e., Temperature (**A**), salinity (**B**), average Chl*-a* (**C**) and size-fractionated chl*-a,* i.e., Chl a-P (pico chlorophyll), Chl a-N (nano chlorophyll) and Chl a-M (micro chlorophyll). Average concentrations of nutrients, i.e., silicates (**G**; SiO_3_), phosphates (**H**; PO_4_), nitrous oxides (**I**; NO_x_), nitrite (**J**; NO_3_), nitrate (**K**; NO_2_) and ammonium (**L**; NH_4_) at the WPO.

Station zonation and associations were relatively dependent on the geophysical positions and directions of currents and eddies^59^. The width of the NEC was reported to be between 8 and 18° N, and that of the NECC was between 2 and 7° N^60^. Kuroshio started after 127° E from NEC bifurcation^31^. Therefore, stations A and B in the present study were associated with the KC (S_KC_) and C-I of the NEC (S_NEC_). Generally, 126.7–128° E is considered the width of the MC, and its undercurrent (MUC) was detected from 400 m below the MC^31^. Additionally, the NEUC existed 200 m below the NEC^60^. Therefore, the sampling area (130°E) is far from the MC, and the sampling depths (0–150 m) were not influenced by undercurrents in the WPO (MUC and NEUC). According to the literatures^31,59,61–63^ and real-time surface geostrophic velocity data, it was found that stations J–N were associated with the ME (denoted as S_ME_), and P and Q were associated with the HE (S_HE_). This is also supported by stations’ cluster analysis (Pearson coefficient) after considering nutrients as variables (Fig. 2C). Furthermore, the position of Station (St.) O (at eddy edge area) was temporarily affected by the NECC (Fig. 1). Despite having an average marginal position between Mindanao and Halmahera (Fig. 1), St. O falls under S_HE_ due to its high TEP_S_, such as that at P and Q (1.7 mD^−1^). They all constitute a substantial contribution to explaining TEP concentrations in the WPO (Fig. 2D). The study transect possessed high temperatures (> 30 °C) with comparatively low salinity levels (< 34.5 psu) at the surface compared to its bottom. However, salinity was relatively high at the MLD of St. C along compared to the NEC than other stations with low temperature. Temperature (Fig. 3A) and salinity (Fig. 3B) were randomly stratified at stations J to N through the upwelling zone of the cold Mindanao eddy (Fig. 1).

### Nutrients and Plankton composition

**Figure 4:**
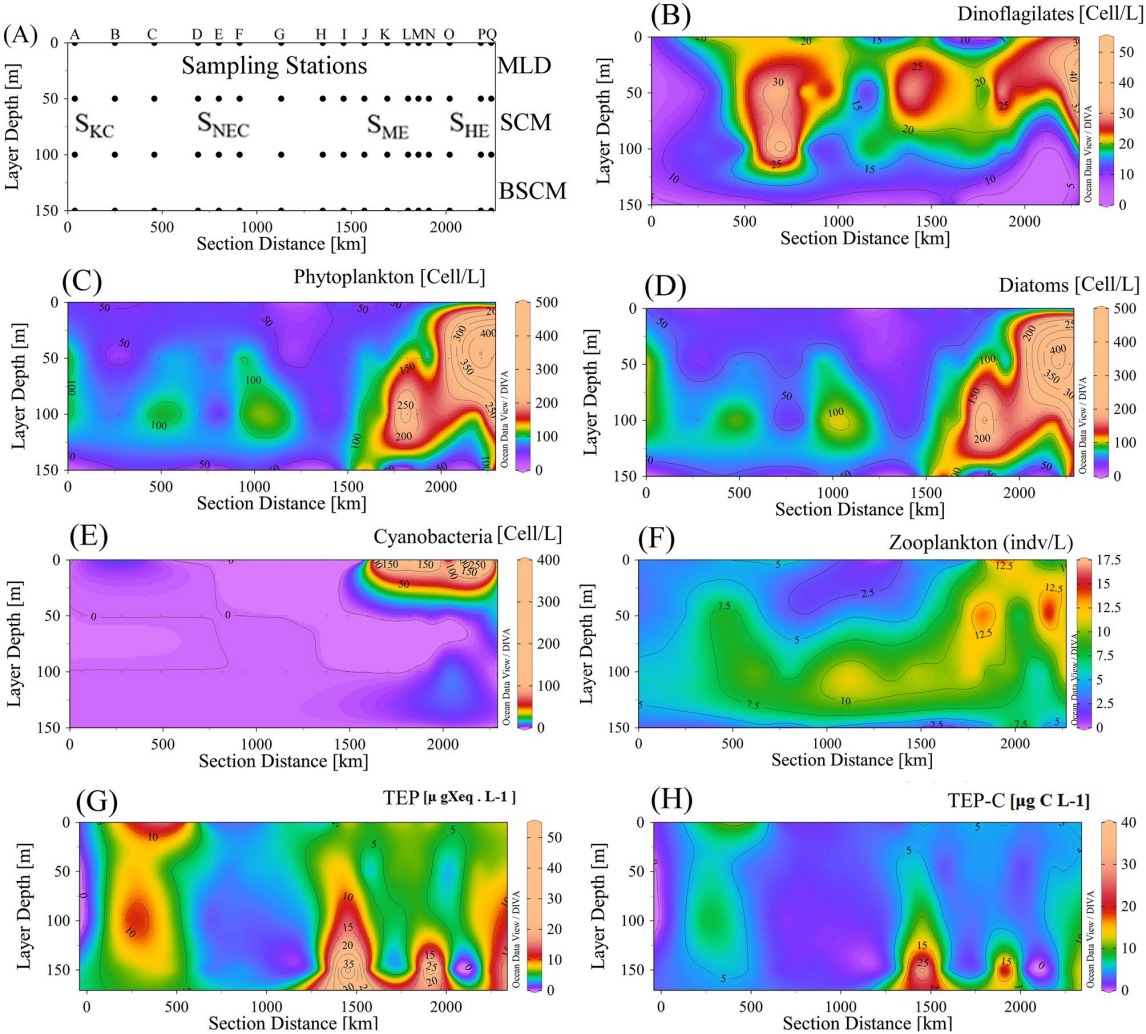
Segmentation of the water column, i.e., the MLD (mixed layer depth), SCM (subsurface chlorophyll maximum) and BSCM (below SCM), with station associations via geophysical forcing, i.e., S_KC_ (stations at the Kuroshio current), S_NEC_ (stations at the Northern Equatorial current), S_ME_ (stations at the Mindanao Eddy) and S_HE_ (stations at the Halmahera Eddy) throughout the study area (**A**) and vertical concentrations of biotic parameters, i.e., diatoms (**B**), dinoflagellates (**C**), cyanobacteria (**D**), TEP (**E**), TEP-C (**F**), zooplankton (**G**), and phytoplankton (**H**), in the WPO.

The average phytoplankton communities were composed of 70% diatoms, where 16% were cyanobacteria and 14% were dinoflagellates. The dominant diatoms were *Nitzschia* sp.*, Cerataulus smithii, Proboscia alata, Nitzschia palea, and Nitzschia filiformis.* Dinoflagillates, i.e., *Pyrophacus horologium, Gyrodinium spirale, Prorocentrum lenticulatum*, and cyanobacteria, *i.e., Trichodesmium hildebrandti* and *T. theibauti*, were also common in the WPO. Analysis showed that the average Chl*-a* was high in the SCM layer of stations M-Q (Fig. 3C). However, a higher chl*-a* content was found below the SCM of stations C-G (Fig. 3C) due to the higher abundance of dinoflagellates (Fig. 4B). However, greater abundances of Chl*-a* P (Fig. 3D) and chl*-a* N (Fig. 3E) were observed at the SCM of stations O, P and Q due to the activity of warm HEs (Fig. 1), with high chl*-a* M at the MLD (Fig. 3F). The high abundance of phytoplankton (Fig. 4C), especially diatoms (Fig. 3D) and cyanobacteria (Fig. 3E), may be liable for these scenarios. In addition, the abundance of zooplankton was found to be higher at the surface of S_ME_ and at the BSCM of S_NEC_ (Fig. 4F), which may also indicate phytoplankton availability in these zones.

The contents of all nutrients (except NO_4_) were higher at stations G to J (Fig. 3) at the MLD. Stations O, P and Q possessed dense NO_2_ concentrations throughout their whole area (Fig. 3K), especially at the subsurface area. Ammonia (NH_4_) was found to be higher at the surface of stations B, C and D (Fig. 3L). The highest concentrations of nutrients (PO_4_, NO_x_, NO_3_ and SiO_3_) were found below the SCM at stations I to Q (Fig. 3).

### TEP concentration and distribution

**Figure 5:**
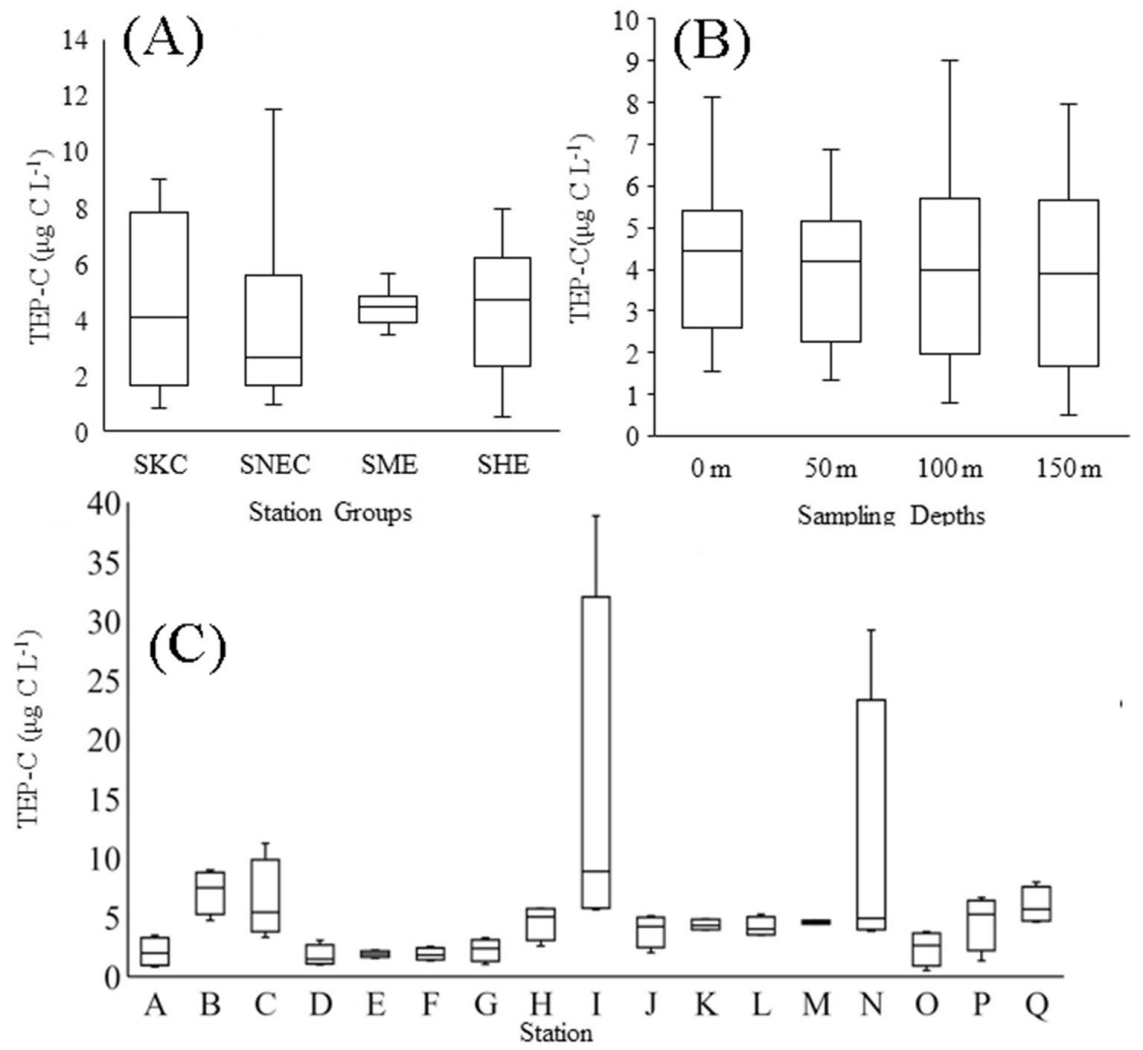
Average variations in TEP_C_ concentrations at all water depths (**A**) and sections (**B**) and at each sampling station (**C**) in the WPO.

The concentration of TEPs was found to be 6.59 ± 7.52 μg Xeq. L^−1^ on average throughout the WPO. The highest average concentration was 20.72 μg Xeq L^−1^ at St. I and lowest average concentration was 2.29 μg Xeq L^−1^ at St. D. It was also found to be higher at 150 m depth at stations I, J, M and N (Fig. 4G). The average horizontal TEP distribution was higher (Fig. 5A) within the S_ME_ area (7.37 ± 7.5 μg Xeq L^−1^) and lower across the S_HE_ (5.76 ± 2.3 μg Xeq L^−1^) than in other areas (Table 1). Vertically, the TEP concentration was higher at the BSCM of S_ME_ (12.29 ± 14.91 μg Xeq L^−1^) and at the MLD of the S_KC_ (7.13 ± 5.21 μg Xeq L^−1^). Average calculations also supported these patterns (Fig. 2D). The highest TEP concentration was found at 150 m at St. I in the NEC (51.80 μg Xeq L^−1^), and the lowest concentration was 0.69 μg Xeq L^−1^ at 150 m in St. O. At the SCM, S_HE_ possessed a higher TEP concentration (6.32 ± 2.45 μg Xeq L^−1^) and a lower concentration at the bottom (BSCM) layer (4.36 ± 5.45 μg Xeq L^−1^) than the other stations (Table 1). S_NEC_ continued to have the lowest TEP concentrations at the MLD (5.27 ± 4.66 μg Xeq L^−1^) and SCM (4.98 ± 3.71 μg Xeq L^−1^) during the sampling periods.

**Table 1:** Variations in the average TEP concentrations and sinking rates with standard deviations across different sea regions during the study.

Data	Water layers	Station groups				
		S_KC_	S_NEC_	S_ME_	S_HE_	H. Ave.*
TEP (μg Xeq. L^−1^)	MLD	7.13 ± 5.21	05.27 ± 04.66	06.37 ± 00.96	6.03 ± 0.89	6.2 ± 02.9
	SCM	6.00 ± 5.41	04.98 ± 03.71	05.41 ± 01.10	6.32 ± 2.45	5.7 ± 03.2
	BSCM	5.46 ± 1.18	10.81 ± 18.26	12.29 ± 14.91	4.36 ± 5.45	8.2 ± 10.0
	V.Ave.**	6.14 ± 4.13	06.51 ± 09.59	07.37 ± 07.50	5.76 ± 2.30	6.4 ± 05.9
TEP_S_ (m.D^−1^)	MLD	0.94 ± 0.20	1.06 ± 0.37	1.53 ± 0.26	1.46 ± 0.24	1.2 ± 0.3
	SCM	1.05 ± 0.14	1.07 ± 0.29	1.57 ± 0.22	1.53 ± 0.30	1.3 ± 0.2
	BSCM	1.09 ± 0.26	1.06 ± 0.30	1.36 ± 0.33	1.73 ± 0.56	1.3 ± 0.4
	V.Ave.**	1.03 ± 0.17	1.07 ± 0.30	1.51 ± 0.26	1.56 ± 0.35	1.3 ± 0.3

*Horizontal average, **Vertical average.

Furthermore, TEP_C_ showed a stratification gradient similar to that of TEP due to its association and relation with TEP_color_ (Fig. 4H). Horizontally, the zonal average TEP_C_ was 4.94 ± 5.64 μg C L^−1^ (1.71–15.53 μg C L^−1^), with the lowest concentration at St. O (0.51 μg C L^−1^) and highest at the BSCM of St. I (38.85 μg C L^−1^). The SCM layer, especially that at a depth of 50 m, possessed lowest average TEP_C_ (3.83 μg C L^−1^) compared to other vertical layers (0 m, 100 m and 150 m). The ranges or distribution patterns of TEP_C_ varied greatly at a depth of 100 m. Moderate variability of the TEP_C_ distribution was found at 0 m with a higher median (Fig. 5B). Variations in TEP_C_ abundances were found at S_NEC_ (Fig. 5C).

### TEP sinking rates throughout the WPO

**Figure 6:**
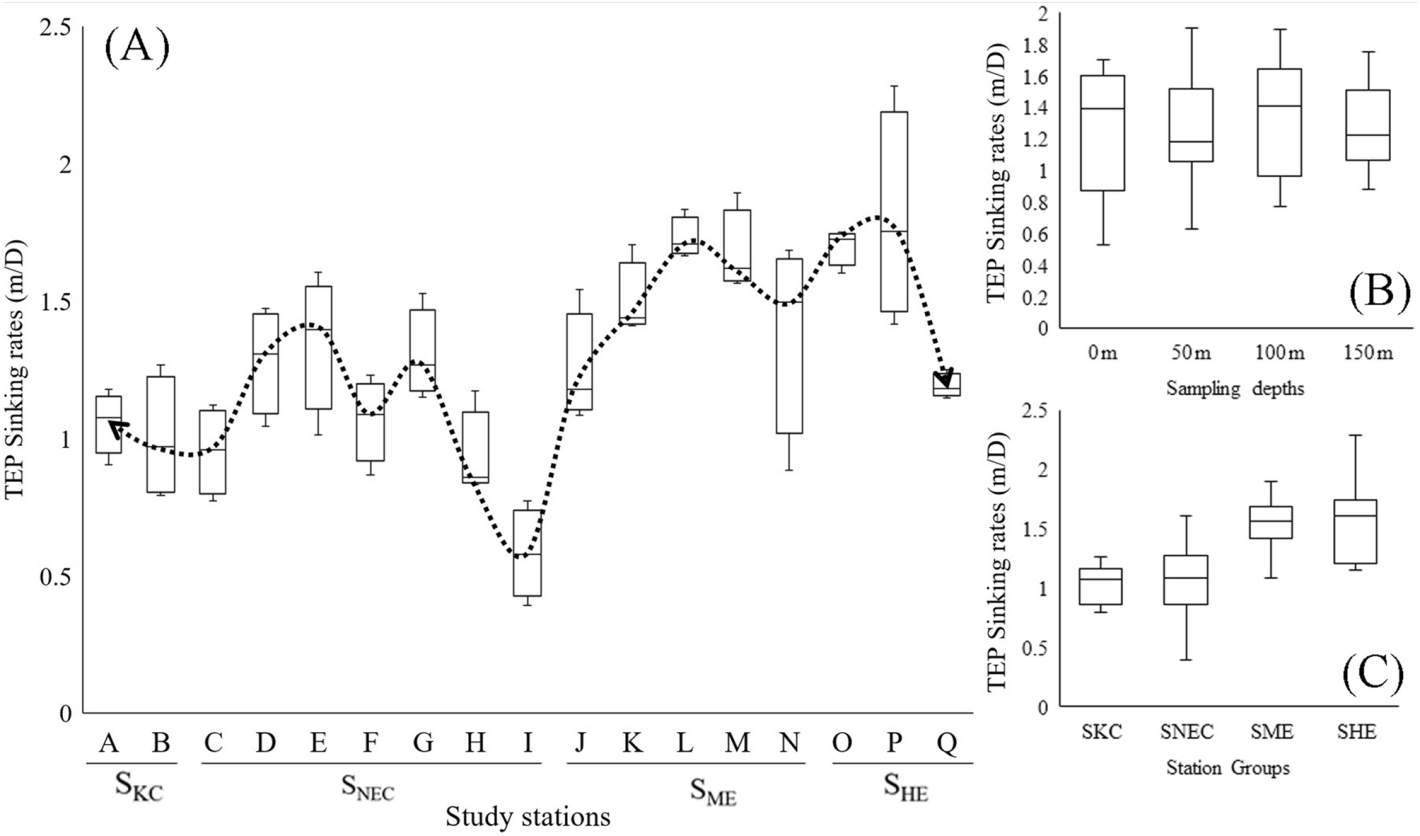
Average variation in sTEP through all sampling stations with line fluctuations (**A**) at each water depth (**B**) and in each section (**C**) in the WPO.

The sinking rates of TEPs (sTEP) were determined using the SETCOL method on the deck of the research vessel. The average sTEP was 1.28 ± 0.37 mD^−1^, and the highest sedimentation rate was at 150 m at station P (2.28 ± 1.5 mD^−1^) in Halmahera due to the combined effects of the NECC (Fig. 1) and the clockwise anticyclonic rotation of HEs^10,62^. However, the lowest sinking rate was found at 150 m at St. I (0.39 mD^−1^) as a result of the ME (Fig. 6A) due to the anticlockwise cyclonic upwelling associated with Mindanao. Depthwise (0, 50, 100 and 150 m) average sinking rates were higher at 100 m (1.32 ± 0.98 mD^−1^), with ranges of 0.77–1.89 mD^−1^, and lower at 0 m (1.26 ± 65 mD^−1^), with ranges of 0.53–1.7 mD^−1^ (Fig. 6B). Considering the water layers, the average sinking rates were higher at the SCM (1.30 mD^−1^) than at both the MLD (1.26 mD^−1^) and BSCM (1.27 mD^−1^). Considering the vertical segments (Fig. 6C), the average sinking rates were found to be higher at S_HE_ (1.56 ± 0.35 mD^−1^), especially at the BSCM (1.73 ± 0.56 mD^−1^), than at all other sections, i.e., S_ME_ (1.51 ± 0.26 mD^−1^), S_NEC_ (1.07 ± 00.3 mD^−1^) and S_KC_ (1.03 ± 0.17 mD^−1^). However, S_ME_ possessed higher vertical sinking rates at both the MLD (1.53 ± 0.26 mD^−1^) and SCM (1.57 ± 0.22 mD^−1^) than at the other stations (Table 1). A low sinking rate was observed at both the MLD (0.94 ± 0.2 mD^−1^) and SCM of the Kuroshio (1.05 ± 0.14 mD^−1^) as well as at the BSCM of the equatorial current (1.06 ± 0.3 mD^−1^).

### Environmental correlations among parameters

**Figure 7:**
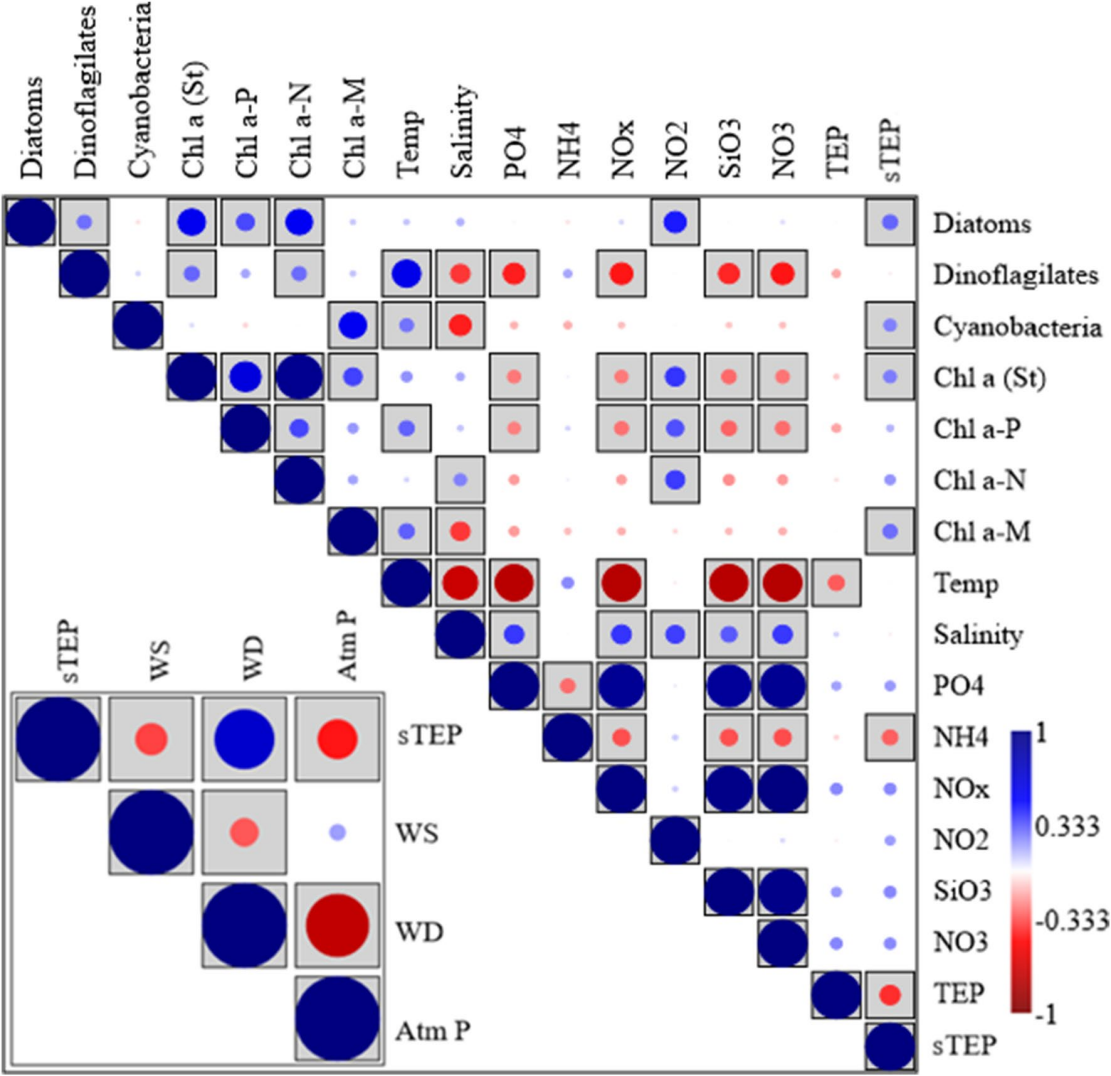
Correlation plots of TEPs and sTEP with other biotic and abiotic parameters after using Pearson’s analysis. The gray box indicates a significant value of P (< 0.05) (*WS* wind stress, *WD* wind direction and *Atm P* atmospheric pressure).

**Figure 8:**
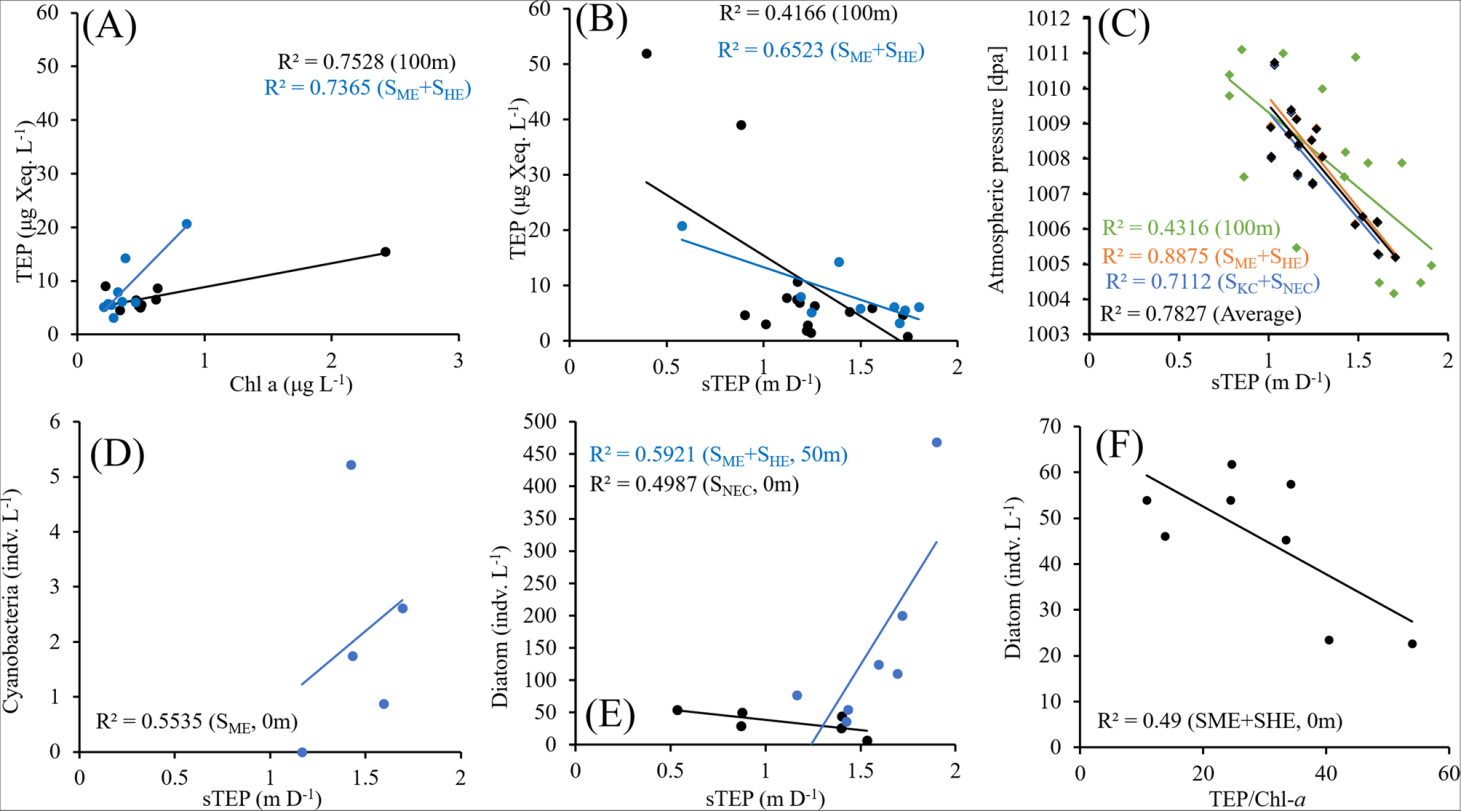
Significant linear regressions of diatoms, cyanobacteria, TEP, TEP/Chl-a ratio and sTEP.

**Figure 9:**
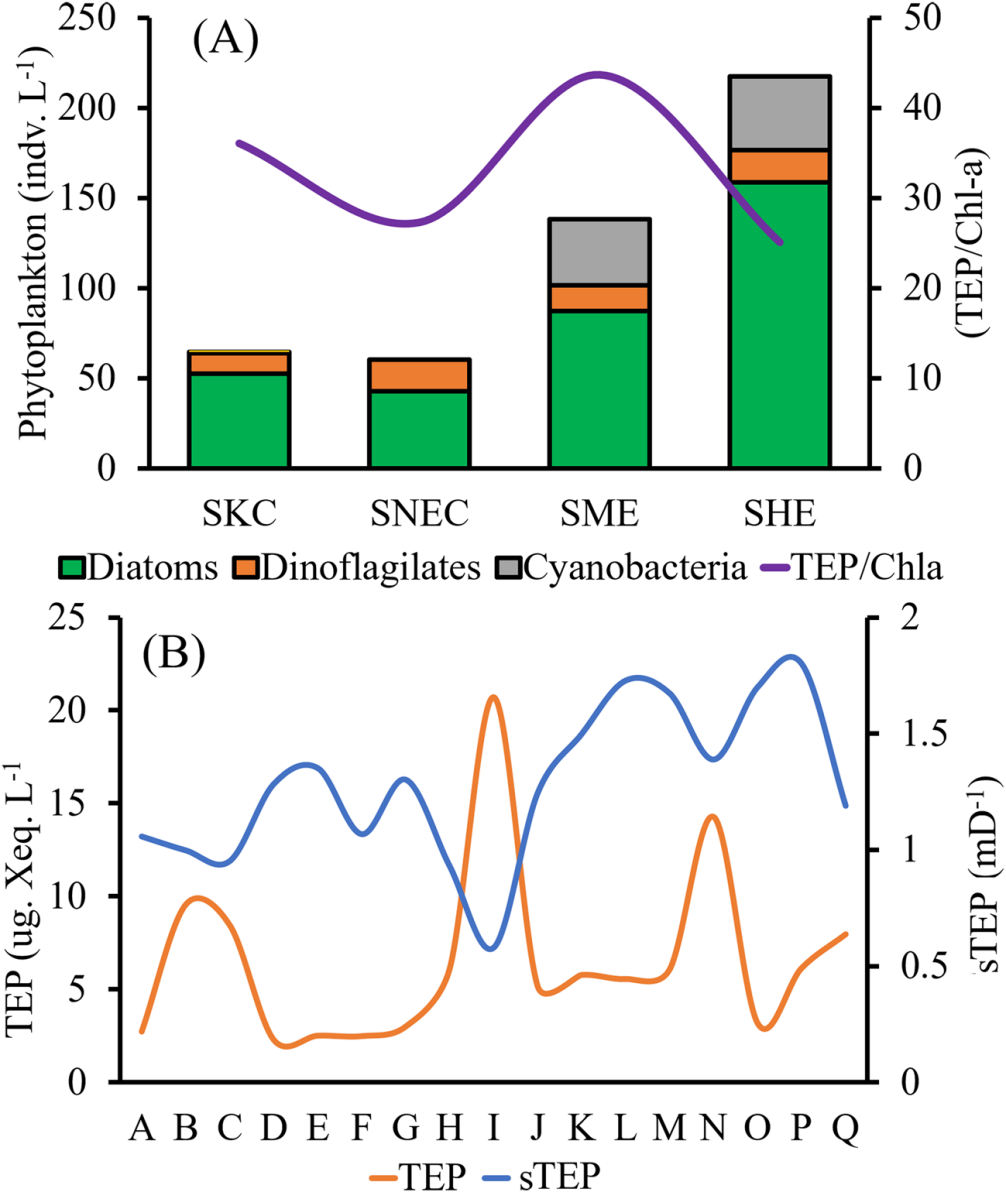
Average phytoplankton assemblages and patterns of TEP/Chl*-a* ratios (**A**) and a comparative line graph of TEP concentrations and TEPs (**B**) in the WPO.

Correlation plots demonstrated the significant correspondences (P < 0.05) of TEPs and sTEP. TEP concentration showed significant negative correlations with temperature (Fig. 7). It showed positive linearity with chl-*a*, especially at 100 m depth at S_ME_ and S_HE_ (Fig. 8A). However, it maintained negative linearity with sTEP in the same zone at similar depths (Fig. 8B), which is also supported by correspondence analysis (Fig. 7). On the other hand, sTEP demonstrated significant positive correspondences (P < 0.05) with diatoms, cyanobacteria and Chl-a (Fig. 7) and negative correspondences with ammonium (Fig. 7). It also showed a highly significant negative correspondence with atmospheric pressure (Atm P) and maintained significant negative linearity (R^2^ = 0.4 ~ 0.8) in the regression graphs, especially at 100 m of the WPO (Fig. 8C). Under the SME, sTEP showed positive linearity with cyanobacteria (R^2^ = 0.55) at 0 m (Fig. 8D) and with diatoms (R^2^ = 0.59) at 50 m (Fig. 8E). In this study, the TEP/Chl-a ratio was positively correlated (R^2^ = 0.49) with diatoms (Fig. 8F) with high amplitudes (Fig. 9), especially at the surface (0 m) of the eddies (S_ME_ + S_HE_).

**Figure 10:**
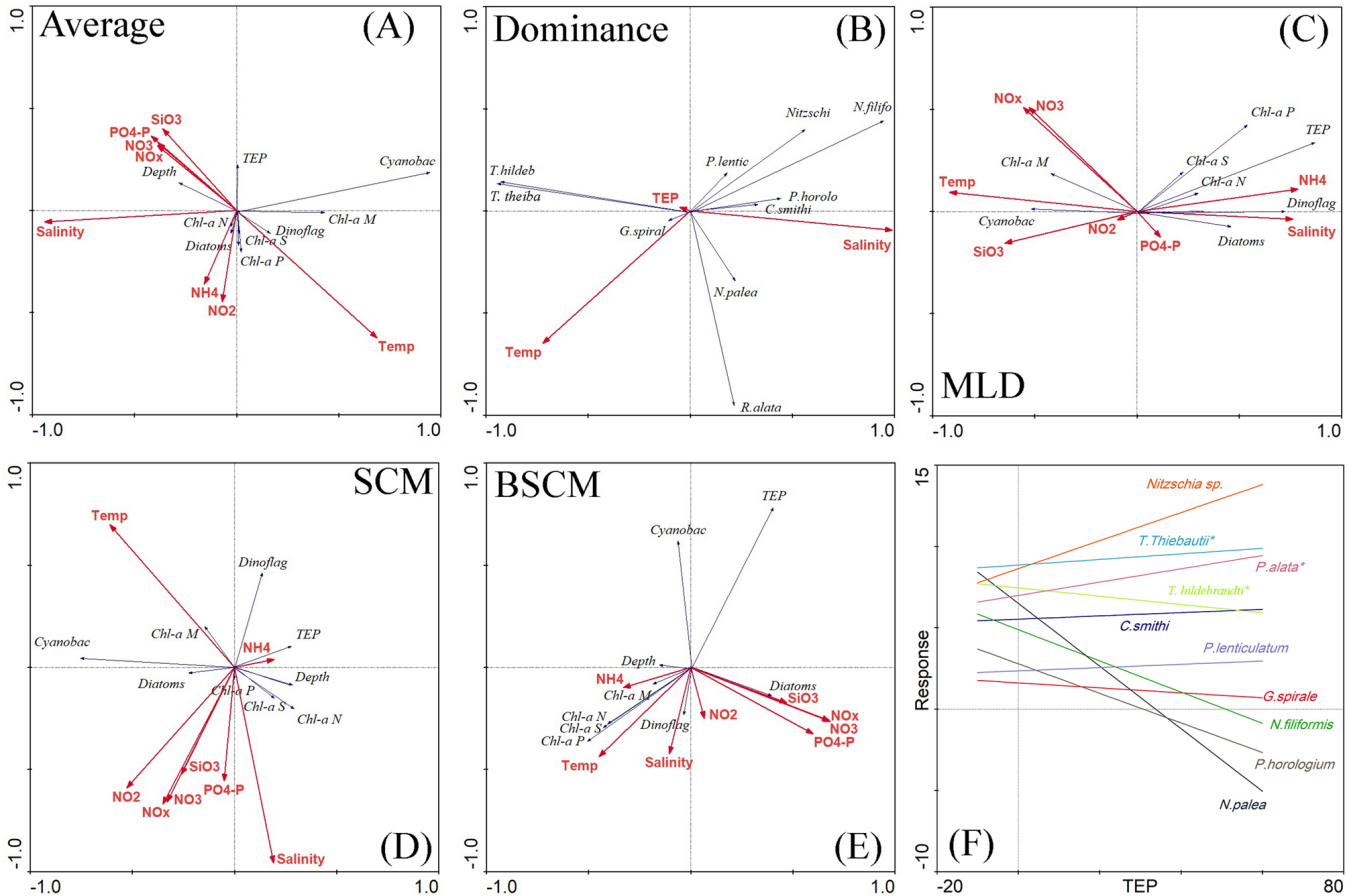
Canonical correspondence analysis (CCA) among different parameters on average (**A**) for the dominant species (**B**) and at different water layers, i.e., MLD (**C**), SCM (**D**) and BSCM (**E**) of the WPO. Response curves of species to TEPs (**F**) with significance levels (* = P < 0.05) after applying a generalized linear model (GLM).

CCA showed the close correspondence of TEP concentration with cyanobacteria abundances and nutrient concentrations (Fig. 10A). Cyanobacteria, i.e., *T. hildebrandti* and *T. theibauti* were more closely related to TEP concentration than the other dominant phytoplankton groups (Fig. 10B). Except for Chl*-a* M, the rest of the size-fractionated chl*-a* concentrations demonstrated correspondences to TEP concentration at the MLD, which may have demonstrated the resemblances of pico- and nanophytoplankton (Fig. 10C). TEP concentration showed close correspondences with NH_4_ contents and dinoflagellate abundances at the SCM (Fig. 10D). Only cyanobacteria demonstrated a close correspondence with TEP concentration at the BSCM (Fig. 10E) compared with other hydrobiological parameters. Using a generalized linear model (GLM), it was found that the responses of *P. alata* (P = 0.02) and *T. theibauti* (P = 0.04) curved positively and *T. hildebrandti* curved negatively (P = 0.03) towards the TEP concentration (Fig. 10F)*.*

## Discussions

### Hydrological stratification of study transects

The Western North Pacific (WNP) summer monsoon was identified in October^64^, and boreal winter was reported between November-December^31^. Therefore, the sampling period was denoted as the transitional time of WNP weather, namely, Autumn^65^. This weather is influenced by ENSO^66^, which drives a critical typhoon season during WPO autumn, i.e., incidence of super typhoon Lan (category 4) on 21 October prior to the 4 days from sampling periods^45^. These northwestern Pacific typhoon intensities brought up water from depths of 75 m and replaced 50% of the water of the mixed layer (30 m) through vertical mixing by wind-driven upwelling^67^. Therefore, the water became well diluted between 0–150 m. These phenomena can help visualize the effects of local geophysical forces (currents, eddies) on environmental parameters across the study transect.

## Biogeophysical influences on TEPs

A number of statistical approaches revealed the significant relation between biogeophysical parameters and TEP concentrations during the current study. For example, salinity can influence TEP patterns^12^. Here, the concentration of TEPs was dense in highly saline areas across the study transect (Fig. 2B). A positive correlation between salinity and TEP mixing intensity was also reported^12^, which was observed at the MLD using CCA (Fig. 10C) during this study. On the other hand, lower salinity levels were found at the MLD than at the BSCM during the present study (Fig. 2B), which is the result of various Pacific Ocean dynamics, i.e., atmospheric convection, precipitation, and evaporation according to the literature^68–70^. Additionally, the NEC brought less nutrient-rich water from the central Pacific^71^, which may cause nutrient deficiencies at S_NEC_ (Fig. 3). Furthermore, nutrient-rich water from the equatorial upwelling zone was prevented by the barrier of the northern boundary convergent front with a weak NECC under the influence of the South Equatorial current (SEC)^72^. This nutrient limitation reduced the biotic production^76^, which may indicate a phytoplankton-derived TEP^4^ availability in the area. Notably, the downwelling feature of Halmahera^62^ combined with these local phenomena may have reduced the nutrient availability at the MLD associated with the eddies (Fig. 5). All of these oligotrophic characteristics coupled with biological phenomena may have influenced TEP depilation at the surface of the WPO.

Moderately high NO_2_ and NH_4_ contents were observed at the Kuroshio (Fig. 3K,L) stations, which were similar to the results of a previous study in summer (considered “before typhoon season”)^42^. This may be liable for the TEP abundances observed at KC (Fig. 4G) due to reasonable phytoplankton diversity^28^ under the influences of nutrients from KC bifurcation from the NEC^73^ and periodic upwelling by cyclonic typhoons^67^. Phytoplankton, especially diatoms (Fig. 4D), were less abundant across the NEC^42^ due to the low nutrient supply coming from the mid Pacific^71^, which drives less TEP production^4^ during autumn (of this study). Cyclonic eddies brought up nutrients from the bottom layer^74,75^, which caused phytoplankton dominance^76^ at the MLD and SCM of the eddies (Fig. 3C). These relations may have induced high TEP concentrations in Mindanao (Fig. 2D), derived from phytoplankton^4^. Nitrogen-based nutrients were higher at 150 m in Mindanao (Fig. 3I), which may also be liable for the high TEP concentrations observed along this upwelling area^27^.

Higher TEP concentration was reported in the Ross Sea (4335 μg Xeq L^−1^) than in other reports (Table 2) due to its high nutrients and productivities^77^. Considering water layers, the MLD of the Adriatic Sea and highly saline Arabian Sea demonstrated high TEP concentrations^23,78^, among the other reported seas (Table 2). It has also been reported that seas condense with higher TEPs than straits, excluding estuaries^79–81^. Among estuaries, the MLD of the Newson Estuary possessed a higher TEPs than others^12,25,82^. However, across the oceans, the MLD of the WPO possessed a lower TEPs than that of the EPO^28^ and Atlantic Ocean^26^. Even the MLD of the northern WPO was reported to have high TEPs^27^ compared to both the MLD and SCM of this study area due to its oligotrophic conditions and low productivities^20^. Considering the BSCM (> 100 m), the TEP concentration was observed to be higher in the WPO than in both parts of the Mediterranean Sea^29,83^. This study hypothesized that active downwelling forces generated by Halmahera^62^ may have brought phytoplankton groups from the surface to the BSCM. Suspended TEPs from upper layers with high sTEP (1.73 ± 0.56 m. D^−1^) can also aggregate TEPs here^24^. Analysis also found close correspondences of cyanobacteria (Fig. 10E), i.e., *T. hildebrandti* and *T. theibauti* with TEP concentrations (Fig. 10B) in this layer. Therefore, high phytoplankton assemblages under the influence of nutrient enrichment through eddy-driven upwelling^61^ and cyclonic activity^67^ induced a high TEP concentration at the BSCM.

**Table 2:** TEPs in different layers of various waterbodies from different studies.

	Sea/area^(references)^	Depths	TEP
		(m)	(μg Xeq. L^−1^)
Seas	The Baltic Sea^14^	Surface layers	145–322
	Eastern Mediterranean Sea^83^	Surface layers	116–420
		50–100	48–189
		100 <	83–386
	Mediterranean Sea^29^	Surface layers	19.4–53.1
		50–100	9.1–94.3
		100 <	4.5–23.5
	Adriatic Sea^78^	Surface layers	4–14,800
	Arabian Sea^23^	Surface layers	507–560
	Weddell Sea^115^	Surface layers	0–48.9
Estuaries and Bays	Jiulong River Estuary^82^	Surface layers	530–720
	Pearl River Estuary^25^	Surface layers	85–1235
	Newson Estuary^12^	Surface layers	805–1801
	Gulf of Cadiz^116^	Surface layers	25–717
	Gulf of Aqaba^117^	Surface layers	130–222
		50–100	106–228
		100 <	23–209
	Chesapeake Bay^118^	Surface layers	37–2820
	Santa Barbara Low Strait^43^	Surface layers	85–252
Oceans	Western tropical North Pacific^27^	Surface layers	43.3
		50–100	42.2
	Eastern tropical North Pacific^28^	Surface layers	22.5
		50–100	9.2
	Eastern subarctic North Atlantic^26^	Surface layers	20–60
	Eastern subarctic North Pacific^28^	Surface layers	28.7
		50–100	11.6
	Western subarctic North Pacific^119^	Surface layers	40–190
	Western Pacific Ocean (This Study)	Surface layers	2.06–14.94
		50–100	1.08–15-33
		100 <	0.69–51.80

### Statistical correspondences of TEP

Western Pacific phytoplankton and microbial communities are controlled by nutrients^46,84^. Therefore, phytoplankton-driven TEPs showed an average close correspondence to nutrients during the present study (Fig. 10A), which has also been found in open seas^27,28^ and polar areas^77^. The surfaces of eddies and upwelling regions also influence particle distribution and organism dominance^85^, which supported the correspondence of Chl*-a* with TEPs at the MLD in both CCA (Fig. 10C) and regression analysis (Fig. 8A) of sampled data as well. Nutrient uptake by phytoplankton^6^ or nutrient upwells from the bottom^74,75^ may be influential under these scenarios.

Diatoms, especially its bloom conditions, were reported as a factor driving high concentration of TEP^15,16,86^. In support of this, a significant positive response curve between TEPs and diatoms (Fig. 8), i.e., *P. alata*, was observed during GLM analysis (Fig. 10F). Chl-a (Fig. 8A), especially Chl*-a* P, showed a significant relation with TEP concentration at the MLD of the study area (Fig. 10C), which is supported by some reports on picophytoplankton, i.e., *Prochlorococcus marinus*, *Anabaena flos-aquae*^17^ and *Synechococcus elongatus*, which are denoted as active TEP sources^7^. On the other hand, the TEP/Chl-a ratio was negatively correlated (R^2^ = 0.49) with diatom abundance (Fig. 8F). It was found to be higher (33.23 ± 21.46) than that observed in terrestrial areas^87^ and lower than that in gulf areas^23^. Additionally, diatoms showed close correspondence (P < 0.05) with chl-a (Fig. 7). This indicates the influence of diatoms on high TEP concentrations at areas with low chl-a abundances, which is also supported by previous reports^23^. The present study observed a significant close relation of two micro cyanobacteria, i.e., *T. hildebrandti* and *T. theibauti*, with TEPs, which may act as a potential source of TEPs in the WPO^88^. As an oligotrophic zone with active geophysical circulations^20^, these phytoplankton species may have influenced TEP production across the study area. This result was also supported by a significant linear regression (r^2^ = 0.6) between diatoms and TEP concentrations (Fig. 8E). In addition, copepods graze on TEPs^17,18^, which may explain the TEP depletion (Fig. 4G) along zooplankton-enriched zones across the whole transect (Fig. 4F).

Biological consumption and air–water exchange also influence CO_2_ exchanges in surface water^89^. On this note, vertical Mindanao mixing (Fig. 1) and high plankton concentration at the MLD (Fig. 4C) may cause lower amounts of dissolved organic carbon in the surface water. In addition, an average low TEP_C_ was observed at the surface of the WPO (Fig. 4H). Therefore, TEPc-CO_2_ exchange, via bubble bursting and wave action, decreased^19^. A previous study suggested that TEPs should be considered in the study of particulate organic carbon (POC)^24^ to estimate the contribution of TEPc to the carbon budget^19^. By obtaining averaged POC satellite data (40 μgL^−1^) of the WPO via SatCO_2_ (see supplementary file 1), this study found a 12.35% contribution of TEPc to the POC, which was considerably lower than that of the reported eutrophic zone (30%)^90^. The oligotrophic condition of the WPO may be driving this scenario^20^. Additionally, TEP sinking was revealed as an important pathway of carbon (0.02–31%) due to its significant influence (r^2^ = 0.65) on the TEP_C_ (Fig. 8B), especially in eddies of WPO^24,91,92^. Considering all these phenomena, in situ POC estimations and their relations should be considered in future research to determine the influences of TEP_C_ on the local carbon cycle.

### Variations of TEP sinking rates

Seawater is denser than TEPs (density 0.70–0.84 g cm^−3^), which indicates that pure TEPs will ascend upwards under ballast-free conditions^93^. Therefore, the sinking rates of TEPs can be negative^93,94^. The presence of inorganic and organic substances in seawater makes it difficult for TEPs to be pure^24^. The sticky gel characteristic of TEPs^95,96^ may cause them to aggregate with various detrituses, particles and organisms, i.e., bacteria, phytoplankton and mineral clays^97^, which carry them to downward in water^19^. In the WPO, high phytoplankton contents (Fig. 4C) and high TEP concentrations (Fig. 4G) at Mindanao have significant positive correlations (Fig. 8A; blue line), indicating its stickiness accordingly. This may cause the moderate sinking rates of TEPs along the ME (Table 1). However, a low TEP stickiness was also reported for increasing TEP concentrations with low downward flow^98,99^. High TEPs with comparatively low sTEP and low phytoplankton abundances confirmed this phenomenon at the BSCM of the ME (Table 1). This result is also supported by the significant negative correlation between TEPs and sTEP (Fig. 8B). Anticlockwise ME-driven upwelling may also influence low sTEP by the physical directions of water as well^61^. Furthermore, cyclonic typhoons can temporarily decrease the sinking rates due to upwells of the water column from the deep sea^67^. Due to its periodic activity, it can be ignored. A low phytoplankton abundance and low TEP concentrations with high sTEP were observed at the BSCM of the HE (Fig. 9B). Downwelling phenomenon of anticyclonic Halmahera may contribute to these senarios at S_HE_ with high sinking rates^62^. Therefore, the sTEP was also influenced by characteristic of geophysical circulations and their directions in addition to the TEP influences at the WPO.

**Figure 11:**
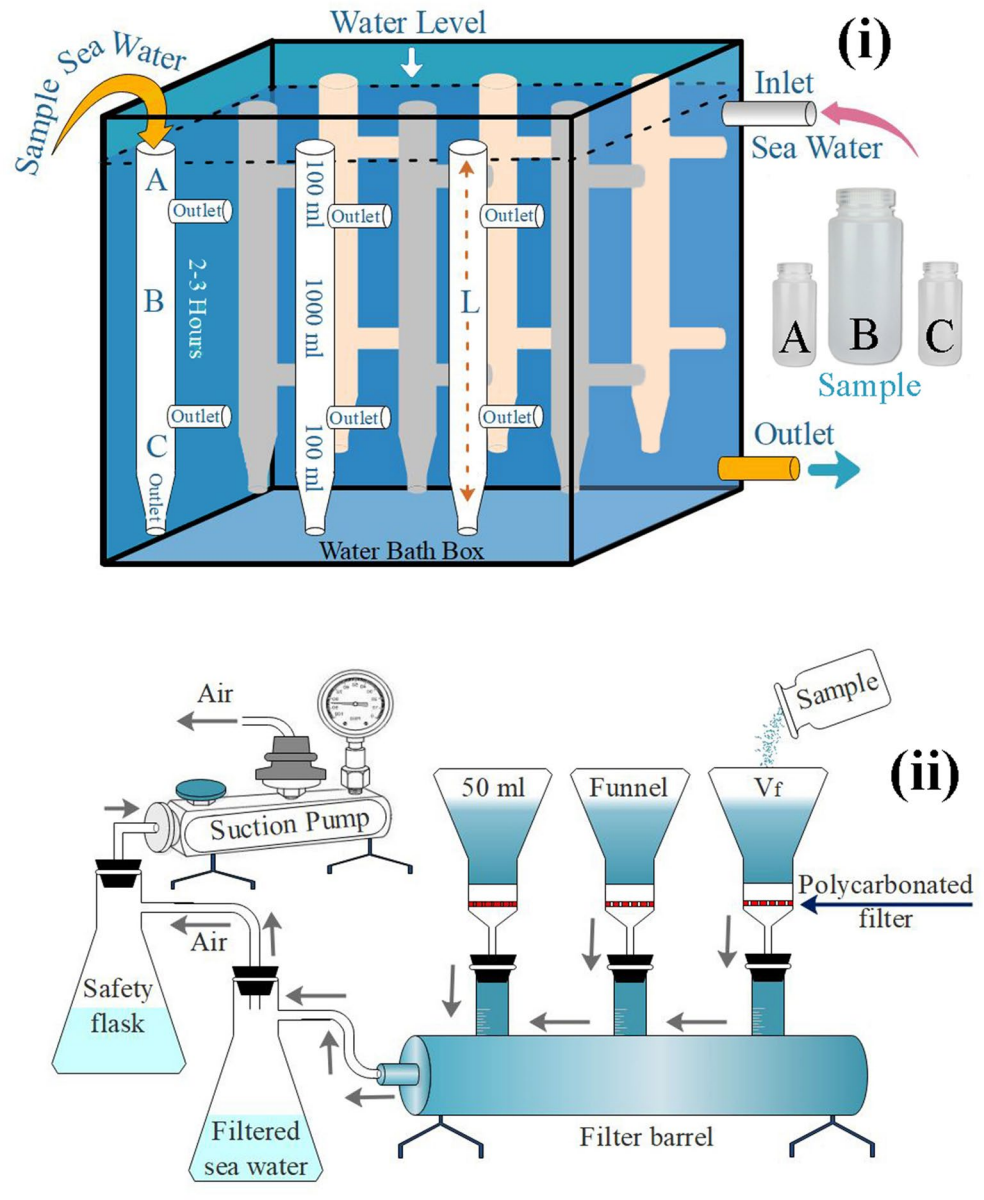
SETCOL setup (**A**) of the vessel and batch filtration processes of colloidal TEP (**B**) according to the literature (Drawn by Dr. M Shahanul Islam, 1st author).

Freshwater lakes have low TEP concentrations with high sTEP (Table 3) due to the influence of phytoplankton cell aggregation^100^. On the other hand, estuaries have also reported high sTEP via SETCOL due to high concentrations of suspended inorganic and organic particulate matter in these areas^24^. On the other hand, the open ocean, i.e., the South Pacific Ocean, demonstrated a low sTEP due to its oligotrophic conditions^94^. TEPs can be affected by variations of sTEP^90^. The SETCOL method^101^ was used to measure the rate of phytoplankton sink^102,103^ as well as TEP sink^24,93,94,104^ due to its simplicity and reliability. The experiment was arranged on the deck of a sampling vessel that collected water from different depths (0–150 m) in separated plexiglass columns (Fig. 11i). It was reported that local geophysical parameters and T-S variabilities (Fig. 2A) caused differences in particle aggregation patterns (Fig. 2B) as well as sinking rates^105^. The significant negative linearity of atmospheric pressure with the sTEP in the WPO may support those reports^105,106^ accordingly (Fig. 8C). However, the turbulence and motion of seawater were ignored in SETCOL on the deck of the sampling vessel, which may have complex effects on particle sinking in the ocean^106,107^. Therefore, this particular situation has remained undefined by theories^24^. It was hypothesized that a high TEP concentration causes a low sTEP due to its porous aggregation structure and that a low TEP with less stickiness causes a high sTEP^19^. Supporting this, the present study observed low sTEP at high TEP concentrations (Fig. 9B) with a significant negative relation (Fig. 8B). All these scenarios indicate a complex relationship between TEPs and sTEP with other environmental parameters, varying from zone to zone across the WPO study transect.

**Table 3:** Variations in TEP_S_ and applied methods from different reports.

Location	TEP_S_ (m d^−1^)	Method
Santa Barbara strait^93^	− 0.22–0.04	SETCOL
South Pacific Ocean^94^	− 0.29 ~ 0.49	SETCOL
Quéntar Lake^104^	1.12 ~ 1.31	Sediment trap
Changjiang Estuary^24^	0.08–1.08	SETCOL
Western Pacific Ocean (This Study)	0.30–2.28	SETCOL

## Conclusions

The current study identified the TEP distribution and its relation with sTEP and other environmental parameters in the WPO. Chl-a significantly influenced the TEP distribution in this oligotrophic region. However, environmental differences in horizontal zones caused diatoms to alter their significant relationships with TEPs, i.e., S_NEC_ (negatively) and S_ME_ (positively). These relations indicated periodical contributions of typhoon-induced mixing at S_NEC_ and upwelling at S_ME_. The nutrient gradient also supported these relational tangents by demonstrating inverse correspondences with zonal biotic parameters separately. On the other hand, atmospheric pressure mainly controlled the sTEP along the whole transect (r^2^ = 0.4–0.8), which may have indirectly influenced the notable negative relation (r^2^ = 0.4–0.6) between TEP concentrations and sTEP. During the study periods, cyanobacteria influenced the TEP increment due to nutrient upwelling, which led to low TEPs at the surface of the S_ME_. It also contributed less to the POC spectra than to the eutrophic zone due to the general oligotrophic environment. Seasonal data collections of TEPs and sTEP can explain the more intense annual relation of particle aggregations in this oligotrophic area in the future. Additionally, the carbon contribution of TEPs under sTEP influences will become picturized more clearly by studying temporal and special variations in particle aggregations at WPOs.

## Materials and methods

### Study area

Water samples were taken from different depths (0, 50, 100 and 150 m) at 17 stations in the WPO (Fig. 1) from 25 October to 12 November (2017) during the Pacific typhoon season^108^. The cruise was conducted near the equatorial area from 2° N, 130° E to 18° N, 126° E. All stations were aligned in straight-lines during sampling to obtain a clear picture of the abundance in a vertically sectioned view (Fig. 4A). Sampling was performed 4 days after the super typhoon “Lan” attack^45^, which experienced maximum sustained winds, reaching 250 km/h (155 mph) throughout the study area (Fig. 1).

### Sample collection

A multiple rosette sampler (MRS with CTD sensors) was used to collect water from four depths (0, 50, 100 and 150 m) at each station. Samples were collected separately to measure phytoplankton abundances, Chl*-a* contents, nutrients, TEP concentrations and sTEP. In a 1 L sampling bottle, phytoplankton samples were collected with 1% formaldehyde for further analysis and identification^24^. For Chl*-a* analysis, the collected seawater was filtered through a 25-mm GF/F and stored at − 20 °C in the dark. Water samples were also taken separately to determine the concentrations of the size-fractionated Chl-*a* spectra, i.e., micro (Chl*-a* M), nano (Chl*-a* N) and pico Chl*-a* (Chl*-a* P) as supporting data. These subsamples were filtered serially through a silk net (20 μm × 20 mm), nylon membrane (2 μm × 20 mm) and Whatman GF/F filters (0.7 μm × 47 mm) for size fractionated Chl-a analysis under a filtration vacuum with less than 100 mm Hg. Seawater from all sampling depths were directly collected in 100 mL sample bottles from MSR chambers via controlled outlets with care and stored at − 25 °C for nutrient analysis.

### Calculation of TEP sinking flux

Edraw Max (v9.4) was used to demonstrate the SETCOL^101^ setup on the research vessel during sampling at each study station (Fig. 11). For observation, three Plexiglass columns (Height = 0.45 m and volume = 1200 mL) for each depth were filled completely with a homogeneous water sample within 10 min after sampling, and a cover was then placed on the setup. In the vessel, the Plexiglass column was kept undisturbed for 2–3 h. A water bath (controlled thermostatically) with water jackets was placed to control the temperature by pumping water around the setup. Settled samples were collected in bottles by successive draining from the upper (A), middle (B) and bottom (C) compartments of those columns (Fig. 11i). The TEP biomass of each segment (A, B and C) from all stations was measured^44^ and calculated according to the following formula:

$${\mathrm{sTEP}}=\frac{{\mathrm{B}}_{\mathrm{s}}}{{\mathrm{B}}_{\mathrm{t}}}\times \frac{\mathrm{L}}{\mathrm{t}}$$

where sTEP is the sinking rate of TEPs; B_s_ is the biomass of TEPs settled into the bottom compartment; B_t_ is the total biomass of TEPs in the column; L is the length of the column; and t is the settling interval. Samples from all depths were triplicated during measurement for better data analysis and marked according to stations for both sTEP and TEPs.

### TEP examination

Samples of TEPs were measured according to the dye-binding method using xanthan gum^43^. A 50–100 mL sample was taken each time (6 replicates) during the colorimetric method after ensuring a xanthan gum curve (fx; as the mean) using absorption measurements. Fifty milliliters (V_f_) of sea water was constantly filtered using a low-pressure (Fig. 11ii) vacuum (150 mm of Hg) using polycarbonate filters (0.4-pm pore-size). Afterwards, particle dying was performed on the filter for ~ 2 s with 500 µL of a 0.02% aqueous solution of Alcian blue (8 GX) in 0.06% acetic acid (pH 2.5). After staining, the filters were rinsed once with distilled water to remove excess dye. Rinsing does not wash off the dye as it binds with substrates^43^. Filters were then transferred into 25-mL beakers with 6 mL of 80% sulfuric acid and soaked for 2 h. The beakers were gently agitated 3–5 times during this period. The maximum absorption of the solution (E_787_) lies at 787 nm, and absorption was observed using a l-cm cuvette against distilled water (B_787_) as a reference. The equation was as follows:

$$TEP\, = \,\left( {{E_{787}} - {B_{787}}} \right)\, \times \,{\left( {V_f} \right)^{ - 1}}\, \times \,{f_x}.$$

The average calibration factor of xanthan gum (f_x_) was measured (9.83) using a regression plot after calculating several absorptions from 0.3–3 ml colloidal free solutions^43^. Carbon contents associated with the TEP concentration (TEP_C_, μg C L^−1^) were calculated after finalizing with the slope (0.75) from the equation as follows^44^:

$$TE{P_C}\, = \,0.75\, \times \,TE{P_{color}}$$

where TEP_color_ is the TEP concentration^24^ in units of μg Xeq L^−1^.

### Measurement of environmental parameters

Temperature and salinity were recorded from different depths by CTD sensors while sampling from the study area. Nutrient (NO_x_, NH_4_, NO_3_^−^, NO_2_^−^, PO_4_ and SiO_3_) analysis was performed by a fully automated (SANPLUS, Dutch SKALAR company) wet chemical analyzer^109^. Samples of phytoplankton (1 L, preserved with 1% formaldehyde) were analyzed according to modified Utermöhl methods^110^ under an inverted microscope after settling for 24 h. The dominance index was used to describe phytoplankton-dominant species using the following equation:

$$Y\, = \frac{n_i}{N} \times \,{f_i}.$$

where N is the total cell abundance of all species, *n*_i_ is the total cell of species *i* and *ƒ*_i_ is the count of occurrences of species *i* in all samples^111^. Chl*-a* was measured from sample water using a fluorescence method in the laboratory after soaking in 90% acetone^112^. The filters were placed into 20-mL glass tubes, and the pigments were then extracted with 5 mL of 90% acetone and stored in the dark at 4 °C for 24 h. After standard calibration, a Turner-Designs Trilogy™ fluorometer was used for chl*-a* measurement.

### Data analysis

Sampling transects were categorized by KC-, NEC-, Mindanao- and Halmahera-controlled water masses in three different layers, i.e., MLD, SCM and below SCM (BSCM). The stations were distributed accordingly (S_KC_, S_NEC_, S_ME_ and S_HE_). After tabulation of the data, various multivariate analyses were performed. Surfer (version 12) was used for the average surface demonstration of all recorded and examined parameters. The concentrations of different parameters were shown using line graphs in Excel stats software and contour color maps in the Ocean Data View (ODV 2018). Particulate organic carbon (POC) data (supplementary file 1) were downloaded from the WGS-84 SOA (Second Institute of Oceanography database). A supplementary color map was produced using SatCO_2_ software (V 3.0) with the SOA data (File name: *NASA_MODIS_MODIS_20171101TO20171130_L3B_GLOBAL_9km_POC_V2017_2020_01_31_15_56_39_High*). Cluster analysis (MCA) was performed after using the Pearson coefficient in Multivariate Statistical Package software^113^. Linear regressions were performed using Microsoft Excel (v2016) software. SPSS (v25) was used for Pearson’s correlation and covariance analyses. Past (v3) software was used to demonstrated the Pearson correlation as graphical plots. Canonical correspondence analysis (CCA) and generalized linear model (GLM) were performed by Canoco software^114^ (version 4.14). Focused sTEP and TEPs data were tested through linear regression against each environmental parameter in Origin Pro (v6). However, data were demonstrated in graphs on the basis of significant relationship accordingly.

## Supplementary Information

Supplementary Information
