# Supplementary material for: Environmental influences on sinking rates and distributions of transparent exopolymer particles after a typhoon surge at the Western Pacific
Source: Sci Rep. 2021 May 31;11:11377. doi: 10.1038/s41598-021-88477-0 (PMC8166891; doi:10.1038/s41598-021-88477-0)
Supplement: Supplementary file 1 — Supplementary Information [file 41598_2021_88477_MOESM1_ESM.docx]

**Title Page**

**Environmental influences on sinking rates and distributions of transparent exopolymer particles after a typhoon surge at the Western Pacific**

M Shahanul Islam^1,3^, Jun Sun^2,3*^, Guicheng Zhang^2,3^, Zhuo Chen^3^, Hui Zhou^4^

1. College of Food Engineering and Biotechnology, Tianjin University of Science and Technology University, No 29, 13^th^ Avenue, TEDA, Tianjin, China
2. College of Marine Science and Technology, China University of Geosciences (Wuhan), Wuhan 430074, PR China
3. Research Centre for Indian Ocean Ecosystem, Tianjin University of Science and Technology, Tianjin 300457, China
4. Key Laboratory of Ocean Circulation and Waves, and Institute of Oceanology, Chinese Academy of Sciences, and Qingdao Collaborative Innovation Center of Marine Science and Technology, Qingdao 266071, China

*Corresponding Author: [phytoplankton@163.com](mailto:phytoplankton@163.com)

Supplementary file 1


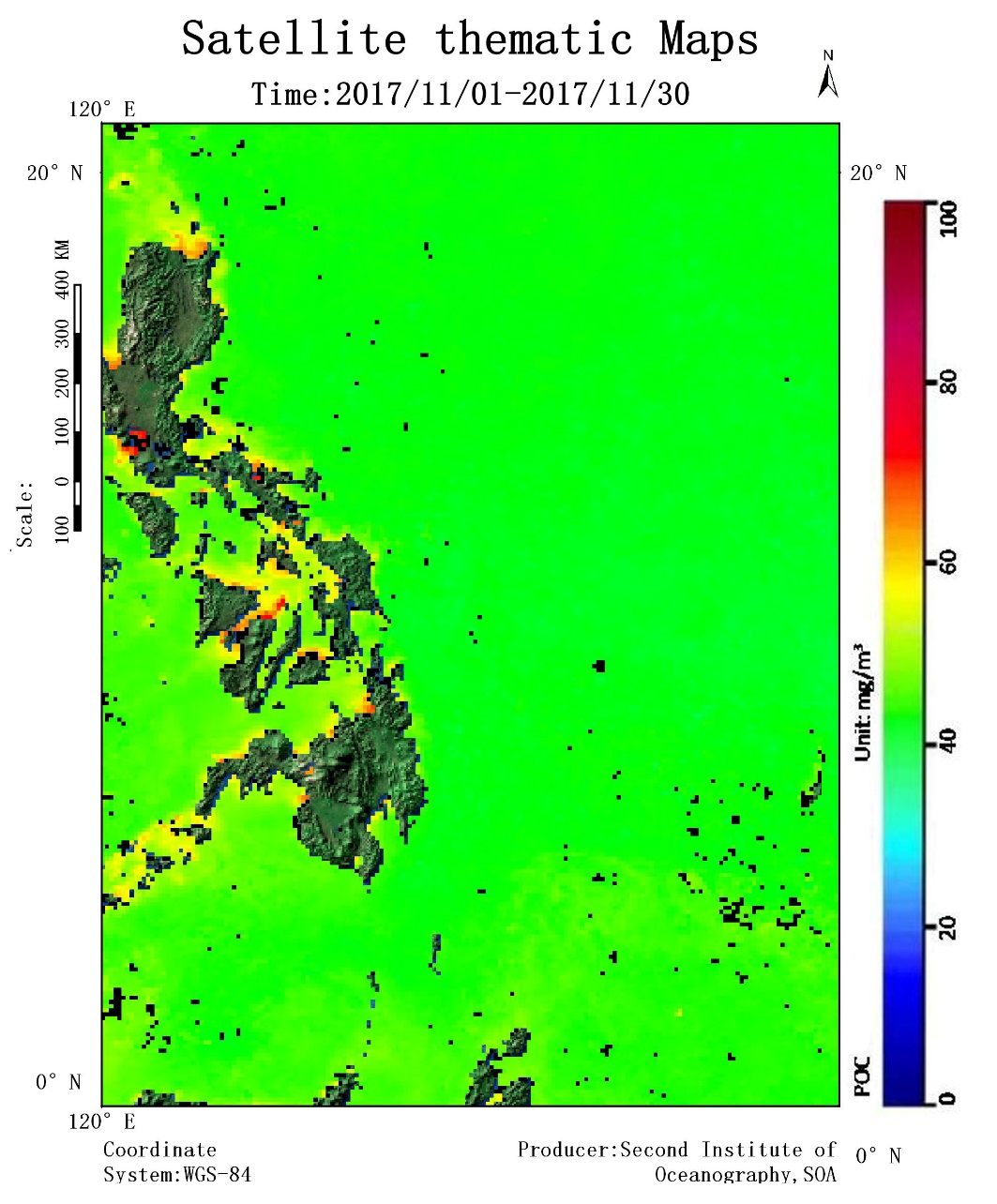


**Supplementary Figure 1:** POC distribution at the study area of WPO
